# Genomic analysis of carbapenem-resistant *Klebsiella pneumoniae* blood isolates from nationwide surveillance in South Korea

**DOI:** 10.3389/fmicb.2025.1562222

**Published:** 2025-05-06

**Authors:** Younggwon On, Jung Wook Kim, Juyoung Lee, Jung Sik Yoo

**Affiliations:** ^1^Division of Antimicrobial Resistance Research, Center for Infectious Disease, National Institute of Health, Korea, Cheongju, South Korea; ^2^Division of Zoonotic and Vector Borne Diseases Research, Center for Infectious Disease, National Institute of Health, Korea, Cheongju, South Korea; ^3^Division of Biobank for Health Sciences, Department of Future Healthcare, National Institute of Health, Korea, Cheongju, South Korea

**Keywords:** antibiotic resistance, whole-genome sequencing, nationwide, surveillance, carbapenem, genomic epidemiology

## Abstract

**Introduction:**

Carbapenem-resistant *Klebsiella pneumoniae* (CRKP) poses a significant threat to public health owing to its multidrug resistance and rapid dissemination.

**Methods:**

This study analyzed CRKP isolates collected from bloodstream infections in nine regions of South Korea using the Kor-GLASS surveillance system between 2017 and 2021.

**Results:**

A total of 3,941 *K. pneumoniae* isolates were collected. Among them, 119 (3%) isolates were identified as CRKP. Most CRKP (79.7%) belonged to sequence type 307 (ST307), followed by ST11 (6.8%). All CRKP isolates exhibited multidrug resistance, with 78.8% carrying the IncX3 plasmid encoding the *KPC-2* gene. Phylogenetic and genomic analyses revealed that ST307 isolates exhibited low single nucleotide polymorphism (SNP) differences. SNP differences among ST307 strains ranged from a minimum of 1 to a maximum of 140, indicating close genetic relatedness. All ST307 strains harbored the KL102 and O1/O2v2 loci, and genomic analysis revealed high prevalence of key resistance genes such as *KPC* (91.5%) and *CTX-M-15* (83.9%), alongside mutations in the *QRDR* (ParC-80I, GyrA-83I) and *ompK* genes. Two major clusters were identified, with cluster 1 harboring yersiniabactin lineage 16 (ICEkp12) and cluster 2 showing higher virulence, including the yersiniabactin lineage 17 (ICEkp10) and colibactin-associated genes.

**Discussion:**

These findings underscore the dominance of ST307 among CRKP isolates in Korea, which is driven by clonal expansion and the critical role of mobile genetic elements. Therefore, enhanced genomic surveillance and targeted infection control measures are urgently required to address the spread of CRKP in clinical settings.

## 1 Introduction

*Klebsiella pneumoniae* is a gram-negative bacterium that can cause a range of infections, including pneumonia, bloodstream infections, and urinary tract infections (Podschun and Ullmann, 1998). The emergence of carbapenem resistance in *K. pneumoniae* is particularly concerning because carbapenems are often considered the last line of defense against multidrug-resistant bacterial infections (Nordmann et al., 2009). CRKP poses a significant threat to hospitals due to its high level of resistance to multiple antibiotics, capacity to cause severe infections (Bratu et al., 2005), and the potential to lead to outbreaks. Previous studies have reported that CRKP bloodstream infections are associated with high mortality rates, ranging from approximately 30%–70%, depending on factors such as antimicrobial resistance patterns, infection severity, and available treatment options (Schwaber et al., 2008). Additionally, CRKP infections frequently result in prolonged hospital stays and increased ICU admissions, further highlighting their clinical burden. *K. pneumoniae* has diverse habitats, including ecological niches in soil, water, and sewage, where it can persist for extended periods under extreme conditions (Pitout et al., 2015). Consequently, this bacterium can survive for prolonged periods in healthcare workers, patients, or hospital environments, potentially serving as a factor in nosocomial outbreaks (Lee et al., 2016).

Carbapenem resistance in *K. pneumoniae* is often caused by the production of various carbapenemases. Although the predominant type of carbapenemase varies by region, carbapenem resistance is commonly associated with carbapenemases, such as *Klebsiella pneumoniae* carbapenemase (KPC), an Ambler class A serine β-lactamase; New Delhi metallo-β-lactamase-1 (NDM-1), an Ambler class B metallo-β-lactamase (MBL); and oxacillinase-48-like (OXA-48-like), which belongs to the Ambler class D β-lactamases. Carbapenemase genes are often located on plasmids and play critical roles in the horizontal transfer of resistance genes. Plasmids, such as IncF, IncN, and IncL/M, frequently harbor carbapenemase genes along with additional resistance determinants and virulence factors, enhancing the adaptability and survival of CRKP in diverse environments. This plasmid-mediated transfer not only facilitates inter-species gene dissemination but also accelerates the emergence of multidrug-resistant strains globally (Carattoli, 2009). Understanding the dynamics of plasmid distribution and their role in the spread of resistance is crucial for effective infection control.

Carbapenem-resistant *Klebsiella pneumoniae* is easily transmitted through clonal dissemination in hospital settings and across the country. Carbapenemase genes are frequently spread globally via the clonal expansion of several successful pathogenic strains (Lee et al., 2016). For example, the global dissemination of KPC-producing *K. pneumoniae*, particularly in the United States and Europe, is predominantly attributed to the expansion of a single dominant strain, ST258. Other globally significant carbapenemase-producing *K. pneumoniae* clones include ST11, ST14, ST101, ST147, ST258, and ST307 (Lee et al., 2016; Wyres et al., 2019).

ST258 has been widely reported in various regions and is associated with high levels of antimicrobial resistance and virulence (Pitout et al., 2015). ST11, another prominent clone, is particularly prevalent in Asia and has been linked to numerous outbreaks and severe clinical cases (Qi et al., 2011). These international clones contribute to the complexity of controlling CRKP infections owing to their ability to spread rapidly across different regions and healthcare settings.

Among the various sequence types of *K. pneumoniae*, ST307 has recently emerged as the predominant type as a result of its widespread distribution and association with high levels of drug resistance (Wyres et al., 2020). This sequence type has been linked to numerous outbreaks and clinical infections worldwide, highlighting its significance in CRKP epidemiology. ST307 has caused significant public health issues in multiple countries with cases reported in Europe, the Americas, and Asia, leading to severe clinical outcomes and complicated infection control efforts (Long et al., 2017). The persistence and spread of ST307 in healthcare settings necessitates ongoing surveillance and targeted interventions to prevent further dissemination (Effah et al., 2020).

In Korea, the major clones produced during the early emergence of carbapenemase-producing Enterobacteriaceae (CPE) were ST11 and ST258 (Yoo et al., 2013). However, between 2011 and 2015, the predominant clones shifted to ST11 and ST307 (Yoon et al., 2018b). Recently, the frequency of ST307, as a major KPC-producing CRKP clone, has increased in some hospitals and regions (Jeong et al., 2022). The Korea Disease Control and Prevention Agency (KDCA) established an antimicrobial resistance (AMR) surveillance system, Kor-GLASS, in 2016 (Lee et al., 2018). This system enables the collection and analysis of data on antimicrobial resistance, providing valuable insights into the epidemiology of resistant pathogens, such as CRKP (Lee et al., 2018). Kor-GLASS has been monitoring nationwide bloodstream infections caused by *K. pneumoniae* in nine regions.

This study aimed to provide a comprehensive analysis of the clinical epidemiology and genomic characteristics of circulating CRKP clones collected by Kor-GLASS in Korea between 2017 and 2021. During this period, Korea experienced a remarkable increase in carbapenem resistant Enterobacteriaceae (CRE) (11,954 cases in 2018 to 23,311 cases in 2021), with 70% of all CRE cases caused by *K. pneumoniae* (Lim et al., 2024). We present the circulating clones and characterize them by analyzing their epidemiological linkage, resistance gene profiles, plasmid distribution, and virulence characteristics. Through this comprehensive approach, we aim to provide actionable insights that could guide public health interventions and improve clinical outcomes in the face of the growing threat posed by CRKP.

## 2 Materials and methods

### 2.1 CRKP isolate and epidemiological data collection

In 2016, KDCA established a nationwide antimicrobial resistance (AMR) surveillance system, Kor-GLASS, in coordination with the Global AMR Surveillance System (GLASS) of the World Health Organization (WHO). The collected data included specimen type, collection date, intensive care unit (ICU) admission status, and isolation region. Only the first isolate from the same patient was included during the collection period. In this study, a total of 119 CRKP isolates were selected from blood-derived strains collected from general hospitals in nine regions of South Korea between 2017 and 2021. All isolates were obtained from hospitalized patients with confirmed bloodstream infections. To ensure accuracy, all CRKP isolates were re-identified before genomic and phenotypic analyses. *K. pneumoniae* identification was performed using automated identification systems such as matrix-assisted laser desorption/ionization time-of-flight mass spectrometry (MALDI-TOF MS). When further confirmation was required, 16S rRNA sequencing was conducted. Among the CRKP isolates collected in Korea between 2017 and 2021, those exhibiting resistance to imipenem or meropenem were selected for whole-genome sequencing (WGS).

### 2.2 Antimicrobial susceptibility tests

All CRKP isolates were tested for susceptibility to 18 antibiotics. The disk diffusion method was used to test susceptibility to 15 antibiotics, including piperacillin, ampicillin-sulbactam, cefazolin, cefotaxime, ceftazidime, cefepime, aztreonam, cefoxitin, amikacin, gentamicin, ciprofloxacin, trimethoprim-sulfamethoxazole, chloramphenicol, tetracycline, and tigecycline. Susceptibilities to imipenem, meropenem, and colistin were measured using the broth microdilution method according to the Kor-GLASS manual. Inhibition zones were measured using calipers, and bacterial resistance traits were determined according to the 2019 CLSI standard guidelines (Clinical and Laboratory Standards Institute [CLSI], 2019).

### 2.3 Statistical analysis

To estimate the statistical significance between CRKP infection status and clinical characteristics, analyses were performed using SAS 9.4 (Statistical Analysis System, version 9.4). Clinical characteristics such as gender, origin, and ICU status were analyzed for statistical significance using chi-square (χ^2^) tests (Table 1).

**TABLE 1:** Demographic and clinical characteristics of patients with blood stream infection with *Klebsiella pneumoniae* (2017–2021).

	No. of isolates (%)		
**Feature**	**CRKP** **[*n* = 119 (3.0%)]**	**non-CRKP** **[*n* = 3822 (96.7%)]**	***P*-value**
**Isolate year**			
2017	6	691	–
2018	12	689	–
2019	7	709	–
2020	34	836	–
2021	60	897	–
**Gender**			0.005
Female	33 (27.7)	1,549 (40.5)	–
Male	86 (72.3)	2,273 (59.5)	–
Origin*			< 0001
Community origin	24 (20.2)	2,832 (74.1)	–
Hospital origin	95 (79.8)	990 (25.9)	–
**ICU**			< 0001
Yes	49 (41.2)	558 (14.9)	–
No	70 (58.8)	3,185 (85.1)	–
**Age**			–
Mean age	69.74	69.97	0.873
(Standard deviation)	(15.82)	(15.35)	–

*Hospital origin: defined as cases with a prior hospitalization duration of ≥ 3 days.

### 2.4 DNA extraction and WGS

All CRKP isolates were first cultured on tryptic soy agar (TSA) plates at 37°C for 18–24 h under aerobic conditions to ensure sufficient biomass for DNA extraction. DNA from each isolate was extracted using the Wizard DNA Purification Kit (Promega Corporation, Madison, WI, United States) with some modifications.

Whole-genome sequencing (WGS) was performed using the Illumina HiSeq sequencing platform following standard short-read, paired-end sequencing protocols. An Illumina HiSeq sequencing library was prepared using the TruSeq DNA (350) nano kit (Illumina, Inc., San Diego, CA, United States).

### 2.5 Genomic assembly and genome analysis

Raw FASTQ sequencing reads from WGS assembly were obtained using SPAdes v3.15.5 (Bankevich et al., 2012) with rasusa v0.6.0 (Hall, 2022) as a random sampler. Antimicrobial resistance genes were annotated using ABRicate v1.0.0 based on the comprehensive antibiotic resistance database v3.2.3 (McArthur et al., 2013) and antibiotic resistance gene-ANNOTation (ARGannot) database release 2019_07 (Gupta et al., 2014). Virulence factor genes were annotated using ABRicate v1.0.0 based on the virulence factor database, release 2023_09. Mobile genetic elements (MGEs) were identified using the ISFinder database release 2020_10 (Siguier et al., 2006). The prophage sequences were identified using DBSCAN-SWA (Gan et al., 2022). To identify the plasmid contigs, we used two different tools: PlasClass v0.1.1 (Pellow et al., 2020) and PlasFlow v1.1 (Krawczyk et al., 2018). Contigs identified as plasmids by both PlasFlow (cut-off = 0.7) and PlasClass (cut-off = 0.7) were considered plasmid contigs, whereas contigs identified from the chromosome or non-plasmid by both PlasFlow and PlasClass were considered chromosome contigs.

### 2.6 Phylogenetic and genomic epidemiology analysis

A core-SNP phylogenetic tree was constructed using kSNP4 (Gardner et al., 2015) and visualized with microreact (Argimón et al., 2016). The Kchooser4 function in kSNP4 calculates the optimal k-mer value, which serves as a key variable for phylogenetic tree construction.

To analyze genomic epidemiology, core genome multi-locus sequencing typing (cgMLST) analysis was performed using SeqSphere+ v9.0.10 (Ridom, Germany) (Jünemann et al., 2013). The cgMLST scheme for *K. pneumoniae* is defined in the cgMLST database (Bialek-Davenet et al., 2014). The number of allelic differences between isolates was used to calculate genetic distances and identify clusters within each sequence type. The resulting data were visualized using a minimum spanning tree in SeqSphere+.

Unlike conventional SNP-based phylogenetic analyses that rely on a single reference genome, we applied an approach in which SNP analysis was conducted within cgMLST-clustered isolates without designating a specific reference genome. In this method, all isolates serve as references against each other, allowing for pairwise SNP analysis across the dataset. This approach generates a comprehensive SNP matrix that represents SNP differences between all isolates, eliminating the influence of a single reference genome selection on the results.

Pairwise SNP analysis was conducted using Snippy v4.6.0, and the genetic relationships among cgMLST-clustered isolates were evaluated. Additionally, Gubbins was used to align single SNPs and predict homologous recombination regions. This analysis produced three SNP scores: the total number of SNPs, the number of SNPs within homologous recombination regions, and the number of SNPs within non-homologous recombination regions.

To define epidemiologically linked isolates within each cluster, we applied a recombination-filtered SNP threshold of five for CRKP isolates. A node-link diagram was generated for isolates with five or fewer SNP differences, where nodes represented isolates from the same year and region. The node size indicates the number of isolates, and the diagram was arranged using a force-directed layout, with the x-axis reflecting the year of isolation. Node distances were set for visual clarity and do not reflect actual biological relationships.

## 3 Results

### 3.1 Clinical epidemiology analysis of *K. pneumoniae* isolates

From 2017 to 2021, a total of 3,941 *K. pneumoniae* (KP) isolates were collected from patient blood, of which 119 isolates (3%) were identified as CRKP (Table 1). This trend was influenced by the increase in participating regions in our surveillance (eight regions in 2017 and nine regions in 2021); however, there was also a noticeable increase within the existing participating regions. In particular, CRKP significantly increased in 2020 and 2021. When examining the distribution by sex, the isolation rates for non-CRKP patients were similar between males and females. However, CRKP was more frequently isolated from males than from females (*p* = 0.005). No significant age differences were observed between the two groups. Analysis of infection types revealed that CRKP was more frequently associated with hospital origin (HO) infections (79.8%), whereas non-CRKP was predominantly associated with community origin (CO) infections (74.1%; *p <* 0.001). Additionally, 41.2% of CRKP cases occurred in patients who had stayed in the ICU, whereas only 13% of non-CRKP cases were associated with an ICU stay (*p <* 0.001).

### 3.2 Characteristics of CRKP isolates

Blood isolated CRKP isolates were collected from nine regions (A–I) in Korea in 2017 (*n* = 6), 2018 (*n* = 12), 2019 (*n* = 7), 2020 (*n* = 34), and 2021 (*n* = 60). CRKP was not identified in regions D and G. Regions with the most isolates were I (*n* = 33, 28.3%), which participated only in 2020, A (*n* = 32, 3%), F (*n* = 24, 20%), and H (*n* = 18, 15%). Interestingly, only four isolates (3.3%) were isolated from the B/C/E regions. *In silico* analysis of CRKP isolates was performed to determine the MLST. A total of nine sequence types of CRKP were identified using this method of analysis. Among these, the most frequently occurring CRKP sequence type was ST307 (*n* = 94, 79.7%), followed by ST11 (*n* = 8, 6.8%) and ST789 (*n* = 4, 3.4%) (Supplementary Figure 1). Among the various STs, only ST307 showed a rapidly increasing trend over the past 5 years. The annual distribution of ST307 isolates showed a significant increase over time, with 1 isolate in 2017 (16.7%), six isolates in 2018 (50.0%), six isolates in 2019 (85.7%), 30 isolates in 2020 (88.2%), and 51 isolates in 2021 (85.0%). This trend highlights the increasing dominance of ST307 among the CRKP isolates in recent years. All CRKP isolates were resistant to penicillin, cephalosporins, monobactams, and fluoroquinolones. Over half of the isolates exhibited resistance to aminoglycosides (64.7%) and tetracyclines (62.2%), whereas the resistance rates for polymyxins were relatively low at 3.4%.

**FIGURE 1:**
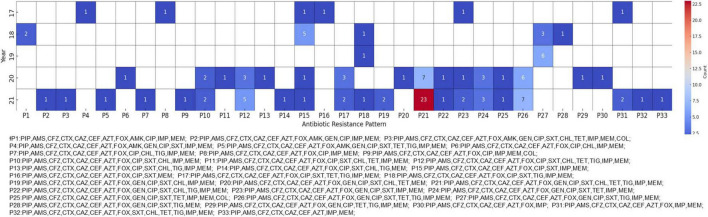
Antibiotic resistance patterns in carbapenem-resistant *Klebsiella pneumoniae* (CRKP) isolates. The heatmap shows the antibiotic resistance patterns of CRKP isolates from 2017 to 2021. Each cell represents the number of isolates exhibiting a particular resistance pattern, with darker red shades indicating higher numbers of isolates. For readability, the patterns are abbreviated and matched to arbitrary code.

To further understand the multidrug-resistant characteristics of CRKP, we analyzed the antibiotic resistance profiles of the isolates. All CRKPs were confirmed to be MDR strains, with increasingly diverse resistance patterns emerging over time. The most prevalent pattern, designated P21, exhibited resistance to antibiotics belonging to the penicillin, cephalosporin, carbapenem, monobactam, aminoglycoside, fluoroquinolone, tetracycline, tigecycline, and sulfonamide classes. The P21 pattern was first identified in 2018 and showed a marked increase by 2021 (Figure 1). This pattern was initially detected in Region A in 2017 and was confirmed to be confined to this region until 2019. However, in 2020, it was detected in provinces B and F. By 2021, the presence of the P21 pattern had been further verified in a hospital in Region I, which participated in the Kor-GLASS surveillance system for the first time.

### 3.3 Genetic and phenotypic characterization of CRKP isolates

**FIGURE 2:**
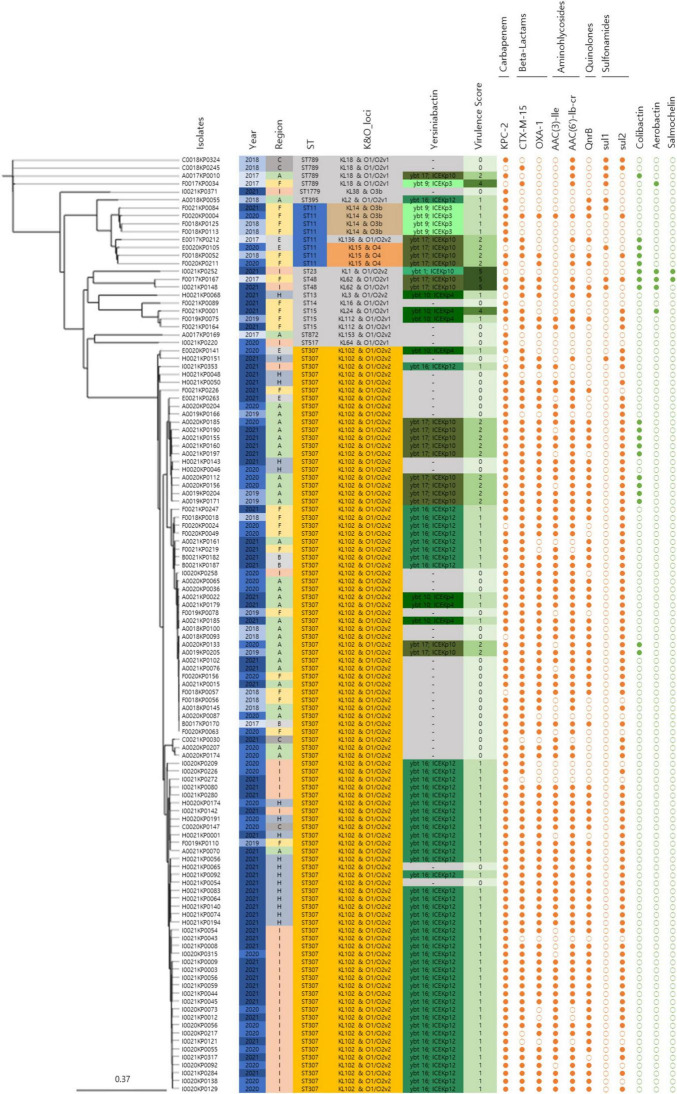
Genomic characteristics and profiles of clustered bacterial isolates. A SNP-based neighbor joining (kSNP4) phylogenetic tree was generated. Information about the year of isolation, region of isolation, MLST type, K&O loci, yersiniabactin type, virulence score, antibiotic resistance genes, and virulence genes is provided.

The major CRKP strains isolated from domestic patients were identified as ST307 and ST11. All ST307 strains consistently exhibited the KL102 and O1/O2v2 loci, whereas ST11 strains had three distinct serotypes: KL14, O3b, KL136, O1/O2v2, KL15, and O4 (Figure 2). In the phylogenetic analysis, the pairwise SNP differences among the ST307 strains ranged from a minimum of 1 to a maximum of 140, with a mean difference of 73.0 and a median difference of 83.0. Compared to the broader SNP difference range observed across the overall strain population (minimum: 1; maximum: 13,689; mean: 4,782.5; median: 111.0), the domestically isolated ST307 strains demonstrated a significantly closer relationship. Notably, the mean SNP difference among the ST307 strains (73.02) was approximately 98.5% lower than the overall mean difference (4,782.5), while the maximum difference (140) was approximately 99% lower than the overall maximum difference (13,689). These low SNP differences indicate a recent common ancestor and suggest that the ST307 strains are closely related in an epidemiological context, strongly supporting their clustering in the phylogenetic tree.

In addition to the SNP-based phylogenetic findings, the CRKP isolates exhibited a high prevalence of major antibiotic resistance genes, highlighting their genomic adaptations to selective pressures. *KPC* (91.5%) and *CTX-M-15* (83.9%) were the most frequently detected genes, followed by *OXA-1* (74.6%). Aminoglycoside resistance-associated genes *AAC(3)-IIe* (61.9%) and *AAC(6’)-Ib-cr* (78.8%) were also prevalent, whereas the quinolone resistance gene *QnrB* was detected in 65.3% of isolates. Sulfonamide resistance genes *sul1* and *sul2* were present in 8.5% and 76.3% of isolates, respectively.

Genomic sequencing revealed mutations associated with antibiotic resistance. Among the 119 isolates, 94.1% (112/119) harbored mutations *ParC-80I* and *GyrA-83I* in the quinolone resistance-determining regions (QRDR) of chromosomal *gyrA* and *parC* genes, underscoring their role in quinolone resistance development. Additionally, all CRKP isolates exhibited mutations in the outer membrane protein genes *ompK36* and *ompK37*, which contributed to resistance against cephalosporins and carbapenems.

IncFII(K) (89.8%), IncFIB(K) (88.1%), and IncX3 (78.8%) were identified as the major plasmid types. Clusters 1 and 2, consisting of ST307 and non-clustered isolates containing various sequence types, showed a similar pattern to the overall major plasmid types. However, Cluster 3, consisting of ST11, exhibited a different pattern, with IncFIA(HI1), IncHI1b(pNDM-MAR), and repB(R1701) each accounting for 66.7%.

### 3.4 cgMLST analysis of CRKP isolates

**FIGURE 3:**
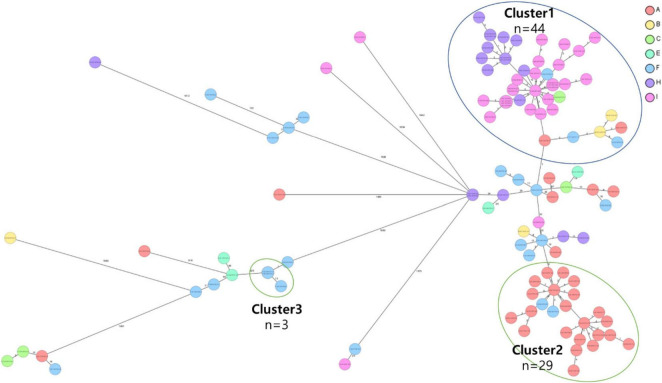
Core genome multi-locus sequence type (cgMLST) analysis of carbapenem-resistant *Klebsiella pneumoniae* (CRKP) isolates from the nine region hospitals in Korea. The isolates were analyzed using cgMLST in Ridom SeqSphere+ and were visualized in a minimum spanning tree. Isolates with 12 allele differences between them were grouped together in clusters with the isolates per cluster shown in circles. The number of different alleles between clusters and unique isolates is shown on the connecting lines (not to scale).

The cgMLST analysis of CRKP isolates revealed the presence of three distinct clusters, highlighting the genetic diversity among the strains. Two of these clusters were primarily composed of the ST307 sequence type, which is recognized as a major CRKP clone and plays a significant role in domestic epidemiology. This finding emphasizes the importance of ST307 in the spread and persistence of CRKP in this region. The third cluster consisted of three isolates belonging to the ST11 sequence type, which is associated with high levels of antibiotic resistance. The identification of these clusters using cgMLST provides critical molecular epidemiological insights into the spread and diversification of CRKP, particularly in the context of domestic sequence types (Figure 3).

#### 3.4.1 Genomic epidemiological analysis of ST307 clusters

Clusters 1 and 2 were identified as the two major groups among the ST307 CRKP isolates, comprising 44 and 29 isolates, respectively. Among the 73 isolates, 67 (91.8%) were found to harbor an IncX3 plasmid containing the *KPC-2* gene, highlighting the significant role of *KPC-2* in the dissemination of CRKP. Both clusters shared highly similar characteristics, including sex, year of isolation, ICU admission status, origin of infection, and antibiotic resistance profile. Comparative analysis of SNPs within each cluster revealed that isolates with five or fewer SNP differences were epidemiologically closely related.

**FIGURE 4:**
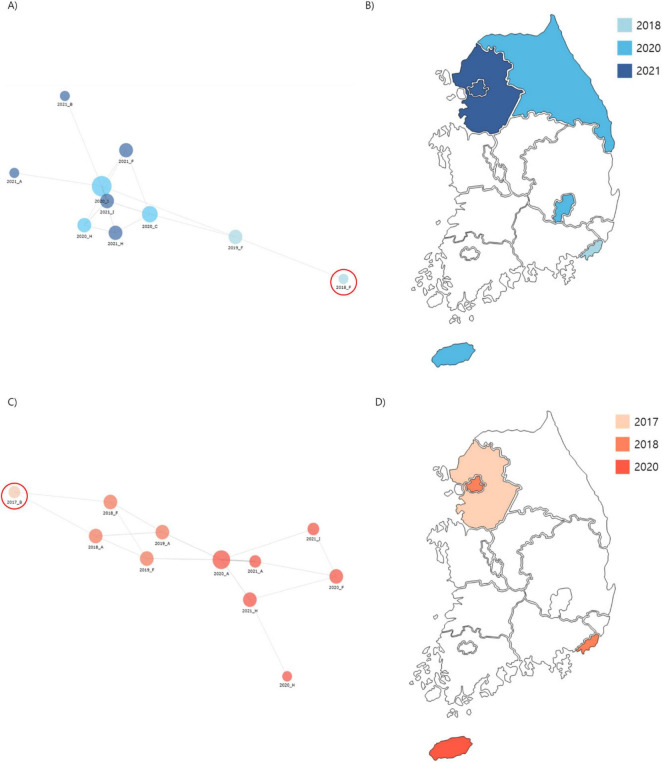
Phylogenetic analysis of carbapenem-resistant *Klebsiella pneumoniae* (CRKP) isolates from ST307 Cluster 1 and 2. **(A)** A core-SNP tree and cgMLST analysis were used to visualize isolate relationships, with a node-link diagram highlighting Cluster 1 proximity. **(B)** Spread routes of strains in Cluster 1 by region. **(C)** A node-link diagram visualizing isolated strains in Cluster 2, with the same layout and parameters as **(A)**. **(D)** Spread routes of strains in Cluster 2 by region, using the same visualization style as **(B)**.

Cluster 1: comprised 44 isolates collected from regions A, B, C, F, H, and I. The first isolate of cluster 1, F0018KP0018, was detected in region F in 2018, and the same clone persisted until 2019 before spreading to other regions (A, B, C, F, H, and I) from 2020 to 2021 (Figure 4). The isolates in Cluster 1 were predominantly distributed in South Korea, and 42 isolates carried the yersiniabactin gene (*ybt*), all belonging to ybt lineage 16, which is associated with the *K. pneumoniae* integrative conjugative element 12 (ICEkp12).

Cluster 2: consisted of 29 isolates collected from regions A, B, F, H, and I. The first isolate of cluster 2, B0017KP170, was identified in region B in 2017. This clone was subsequently detected in regions A and F during 2018–2019 and later spread to regions A, F, H, and I from 2020 to 2021 (Figure 4). The isolates in cluster 2 exhibited a dissemination pattern from metropolitan areas to South Korea.

In terms of virulence, 48% (14/29) of Cluster 2 isolates carried the *ybt* gene. Among these, 11 belonged to ybt lineage 17 associated with ICEkp10, and 3 belonged to ybt lineage 10 associated with the ICEkp4 complex. Furthermore, 11 isolates harbored colibactin-associated genes that induced DNA damage in host cells, significantly increasing their pathogenic potential. These findings indicate that the isolates in Cluster 2 exhibited higher virulence scores than those in Cluster 1, reflecting their markedly increased pathogenicity.

#### 3.4.2 Phylogenetic comparison of ST307 with globally reported genomes

**FIGURE 5:**
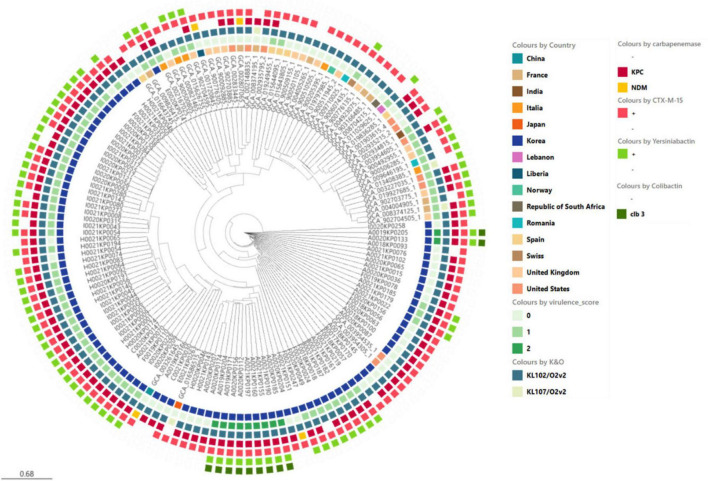
Phylogenetic analysis of ST307 *Klebsiella pneumoniae* isolates including domestic and global reference genomes. The SNP-based neighbor joining (kSNP4) phylogenetic tree was generated. *KPC* gene and *VF* genes are indicated.

We compared the genetic characteristics, virulence factors, and antibiotic resistance genes of ST307 *K. pneumoniae* strains isolated from domestic sources with those of global reference strains. Global reference strains were selected from all available KP strains in the NCBI Assembly database and filtered by ST and relatedness, resulting in a total of 94 domestic isolates and 52 global reference genomes for comparative analysis (Figure 5).

Recent studies have reported ST307 as a predominant CRKP clone in multiple regions, including North America, Europe, and Asia. Wyres et al. (2020) demonstrated the global emergence of ST307, whereas Peirano et al. (2020) identified it as the most frequently occurring ST in Canada. In South Korea, Yoon et al. (2018b) reported ST307 as the dominant CRKP clone, particularly associated with KPC-2-producing strains. These findings highlight the widespread prevalence and clinical significance of ST307.

The domestic Isolates exhibited a relatively high average virulence score of 0.8, whereas the global reference strains had a comparatively lower average virulence score of 0.4. Notably, *ybt 16; ICEKp12* type of Yersiniabactin was frequently observed in domestic isolates, whereas the global reference strains showed a lower prevalence of yersiniabactin with a wider variety of types. Regarding antibiotic resistance genes, *KPC-2* and *CTX-M-15* were observed in both domestic and global strains, but their frequencies were higher in domestic isolates.

#### 3.4.3 Genomic epidemiological analysis of ST11 cluster 3

Cluster 3 consisted of three CRKP isolates collected from region F. The first isolates of Cluster 3, F0018KP0113 and F0018KP0125, were identified in region F on the same day in 2018. No identical clonal strains were detected in 2019; however, a similar clone, F0020KP0004, was identified in the same region in 2020. While the 2018 isolates were closely related, the 2020 isolate exceeded the SNP threshold, indicating genetic divergence over time.

Whole-genome sequencing revealed that all ST11 isolates in Cluster 3 exhibited the same capsular (KL) and O-antigen (O) type: KL14/O3b. Unlike ST307, which consistently displayed KL102/O1/O2v2 regardless of the cluster, ST11 isolates generally exhibited greater serotype diversity. However, the isolates within Cluster 3 consistently exhibited the same serotype, suggesting potential clonal stability within this cluster. All ST11 isolates in Cluster 3 harbored bla_KPC-2 and contained various antibiotic resistance genes. The identified genes were as follows: TEM (66.6%, 2 isolates), CTX-M-15 (33.3%, 1 isolate), OXA-1 (33.3%, 1 isolate).

Additionally, GyrA-83I and ParC-80I mutations in the quinolone resistance-determining region (QRDR) were detected. All three ST11 isolates carried yersiniabactin-related genes (fyuA, ybt, and irp). However, other ICEkp elements, colibactin (clb), and hypervirulence-associated genes (rmpA and iuc) were not detected.

Unlike ST307, which has spread across multiple regions, the ST11 isolates in Cluster 3 were restricted to region F and persisted over multiple years. These findings suggest that the ST11 isolates in Cluster 3 are more likely associated with hospital-acquired infections (HAIs) rather than widespread dissemination.

## 4 Discussion

The first case of CRKP in South Korea was reported in 2010 (Rhee et al., 2010). Since then, interest in CRKP has increased, and its notable rise in incidence has emerged as a major public health concern. According to mandatory national surveillance of CRE, *K. pneumoniae* is the most common species, accounting for 68.1% of all CRE cases. Among these, the KPC type was detected in 76.7% of cases, with the KPC-2 subtype constituting 98.2% of KPC-producing strains (Kim et al., 2023). Several studies have investigated the clonal types, genomic epidemiology, and virulence factors of CRKP strains in South Korea. However, most of these studies have been limited in scope, focusing on specific hospitals or regions (Kim et al., 2010, Lee et al., 2016) rather than providing a comprehensive national analysis. Furthermore, detailed genomic characterization, including the distribution of resistance and virulence genes, remains insufficient (Yoon et al., 2018a). To address these gaps, our study provides a systematic nationwide analysis of CRKP isolates, integrating clonal type distribution, genomic epidemiology, and virulence factor profiling.

Several differences were observed in the clinical data between CRKP and non-CRKP cases. Gender distribution analysis revealed that males were significantly more affected by CRKP than females (72.3% vs. 27.7%). This sex difference may be related to biological, behavioral, or healthcare access factors, indicating the need for further investigation to understand susceptibility differences between the sexes. When the two groups were compared based on infection routes, CRKP was significantly associated with hospital-origin (HO) infections, whereas non-CRKP infections were significantly associated with community-origin (CO) infections. A study conducted in the United States reported that CRKP infections primarily occurred in hospitals and were more prevalent among male patients (Han et al., 2017).

Additionally, 41.2% of CRKP cases occurred in patients who had stayed in the ICU, whereas only 13% of non-CRKP cases were associated with ICU stay, confirming that the ICU is crucial for infection control measures. ICU admission is an important risk factor for CRKP (Wang et al., 2023). Several reports have indicated that the isolation rate of CRKP in the ICU is high. This is attributed to the characteristics of the ICU, including critically ill patients, heavy use of invasive procedures, frequent use of antimicrobials, and the structure that facilitates transmission (Li et al., 2020).

In South Korea, when surveillance for CRE began, *KPC-2 K. pneumoniae* ST11 and ST258 strains were reported (Yoo et al., 2013). However, a study conducted on KPC-producing *K*. *pneumoniae* isolates from 2013 to 2015 found that the proportion of ST307 was 46.2%, while ST11 accounted for 21.3% (Yoon et al., 2018b). In our study, where we investigated bloodstream infections from 2017 to 2021, ST307 was overwhelmingly dominant among CRKP isolates, accounting for 94 (79.7%) of the CRKP blood isolates in our study.

*K. pneumoniae* ST307, a recently developed and successful global clone, has spread worldwide over a relatively short period. First appearing in the 1990s, this clone sequentially acquired fluoroquinolone resistance through *QRDR* mutations and *CTX-M-15* and later continued its regional spread by acquiring carbapenemases, such as KPC and NDM, in various parts of the world. These characteristics have established it as a significant endemic clone in various regions, including Italy, Colombia, the USA (Texas), and South Africa (Shropshire et al., 2022). ST307 carries four types of carbapenemases and has shown an increasing trend in Italy, Romania, Spain, and other countries. The predominant genotype varies by country; Romania and Turkey have NDM, Spain has KPC, and Serbia has OXA-48 (Mabel et al., 2024). ST307 has caused outbreaks in various European countries, including Spain, Portugal, France, Germany, the United Kingdom, and Netherlands. Additionally, it is spreading to Colombia, South Africa, and Italy, replacing ST258 (Peirano et al., 2020).

ST307, the most frequently occurring sequence type in Korea, is broadly distributed across the country without significant regional variation, suggesting that ST307 has established itself as a dominant clone of CRKP in Korea. The widespread presence of ST307 underscores its role in the dissemination and persistence of CRKP, making it a key target for epidemiological investigation. According to domestic research, *K. pneumoniae* ST307, which is highly resistant to fluoroquinolones, was isolated in South Korea in 2013. These strains frequently harbor the *qnr* gene, *aac(6’)-Ib-cr* gene, and *CTX-M-15* gene (Park et al., 2015). Considering their genetic background, the isolates were very similar to the CRKP isolates analyzed in this study, except that they did not carry the KPC gene. Notably, most CRKP isolates harbored the IncX3 plasmid containing the *KPC-2* gene, which likely facilitated extensive dissemination of these isolates. The IncX3 plasmid is a well-known narrow-range plasmid with a high conjugation ability and stability. Its relatively small size (30–60 kb) results in a low fitness cost, which enables its wide dissemination.

Analysis of toxin genes in CRKP isolates revealed a potential link between the presence of virulence factors and antibiotic resistance genes. The concurrent presence of toxin genes, such as ybt (yersiniabactin) and clb (colibactin), along with resistance genes, such as *KPC-2*, suggests that these genetic elements may be co-located on mobile genetic elements (e.g., plasmids) or chromosomal regions. This genetic arrangement can facilitate simultaneous dissemination through horizontal gene transfer, thereby enhancing the virulence and survival of CRKP strains. The presence of toxin genes, such as ybt, in CRKP isolates has been associated with increased pathogenicity, enabling the bacterium to sequester iron through siderophore-mediated mechanisms (Bachman et al., 2011). The coexistence of resistance genes, such as KPC-2, enhances the survival of these strains under selective pressure from antimicrobial use. This synergy between virulence and resistance genes likely contributes to the rapid establishment and persistence of CRKP in clinical and community settings.

Furthermore, studies have reported that ybt and clb are often located on integrative conjugative elements (ICEs) or plasmids that can be transferred across strains or species (Wyres et al., 2020). This genetic mobility underscores the importance of monitoring both virulence and resistance determinants to better understand their roles in the spread of multidrug-resistant organisms. The IncX3 plasmid’s unique characteristics, including its high conjugation ability and stability, make it an effective vehicle for the dissemination of carbapenemase-encoding genes. Although it is known to carry carbapenemases, such as NDM, KPC, and OXA-181, it does not harbor other resistance genes (Lam et al., 2021). As reported by Peirano et al. (2020) it is probable that the current epidemic strain was formed through a sequential process: first, the introduction of fluoroquinolone-resistant and ESBL-producing ST307 into the country, followed by the acquisition of the KPC gene through the transfer of the IncX3 plasmid.

Core genome multi-locus sequencing typing and SNP-based phylogenetic analysis revealed the genetic relatedness of ST307 CRKP isolates, identifying it as the predominant type in Korea. Two major clusters, clusters 1 and 2, were identified with distinct yet overlapping patterns of geographical and temporal dissemination. Cluster 1 originated in region F in 2018 and spread to regions A, B, C, H, and I by 2021, whereas Cluster 2 began in region B in 2017, expanding to regions A and F by 2019 and further to regions H and I by 2021. This type is widely distributed across regions, suggesting a rapid nationwide introduction and dissemination within a short period. In contrast, ST11 isolates in Cluster 3 exhibited a more localized distribution pattern, being restricted to a single region (Region F) over multiple years. However, this does not imply that ST11, as a whole, is confined to a single region, as previous studies have reported the presence of ST11 in various locations. Rather, these findings suggest that, unlike ST307, which rapidly disseminated nationwide, Cluster 3 ST11 isolates may have persisted in a hospital environment over time, leading to their long-term presence in a specific setting.

Genomic epidemiological analyses further highlighted clonal spread and resistance mechanisms of the isolates. Phylogenetic analysis based on core SNPs demonstrated that ST307 isolates had a mean SNP difference of 73 and a maximum difference of 140, indicating a high degree of genetic similarity and supporting the clonal expansion hypothesis. Two major clusters, designated Clusters 1 and 2, were identified using cgMLST analysis. Cluster 1 was initially detected in region F in 2018 and subsequently spread to regions A, B, C, H, and I by 2021, suggesting localized emergence followed by regional dissemination. In contrast, Cluster 2 was first identified in region B in 2017, later expanded to regions A and F by 2019, and spread further to regions H and I by 2021, illustrating a stepwise dissemination pattern. Additionally, isolates with five or fewer SNP differences were classified as “epidemiologically linked strains,” further reinforcing the role of clonal spread in the propagation of CRKP across South Korea. In contrast, ST11 isolates in Cluster 3 exhibited a broader SNP difference range, suggesting greater genetic diversity within this lineage. Additionally, while ST307 isolates consistently exhibited the KL102/O1/O2v2 serotype, Cluster 3 ST11 isolates showed three different capsular and O-antigen types (KL14/O3b, KL136/O1/O2v2, and KL15/O4). This variability in serotypes highlights the genetic heterogeneity within Cluster 3 ST11 isolates compared to the relatively clonal nature of ST307.

These findings suggest the potential for horizontal gene transfer and clonal expansion of antibiotic resistance genes within the domestic environment to facilitate the dissemination and spread of resistant strains. Collectively, our findings indicate that the ST307 *K. pneumoniae* strains isolated in Korea adapted to and spread within the domestic environment by acquiring specific virulence factors and antibiotic resistance genes. The coexistence of toxin genes, such as *ybt* and *clb*, along with resistance genes, such as *KPC-2*, may enhance both the pathogenicity and survival of these strains, contributing to their rapid dissemination. Genomic epidemiological analyses further highlighted clonal spread and resistance mechanisms of the isolates. Using a threshold of five or fewer SNP differences, two primary clusters, Clusters 1 and 2, were identified, each showing distinct yet overlapping patterns of geographical and temporal dissemination.

Despite the robust genomic epidemiological insights provided by this study, certain limitations should be acknowledged. This study primarily focused on CRKP isolates collected from bloodstream infections between 2017 and 2021, limiting the ability to assess long-term trends in genetic evolution and dissemination patterns.

Our cgMLST and SNP-based phylogenetic analyses strongly suggest that ST307 dissemination is primarily driven by clonal expansion, as indicated by the high genetic similarity and minimal SNP variations among isolates within the same cluster. However, we acknowledge that our WGS approach has limitations in fully capturing mobile genetic elements (MGEs) such as plasmids and transposons, which are key drivers of HGT. Due to these constraints, we cannot entirely rule out the possibility that plasmid-mediated HGT contributes to the spread of resistance genes.

Furthermore, the absence of detailed clinical outcome data restricts a comprehensive understanding of the clinical impact and prognosis of CRKP infections. These limitations highlight the need for further research incorporating long-term surveillance, experimental validation of HGT mechanisms, comprehensive clinical data, and advanced sequencing methods (such as long-read sequencing) to better assess the role of MGEs in ST307 dissemination and enhance infection control strategies.

## Data Availability

The datasets presented in this study can be found in online repositories. The names of the repository/repositories and accession number(s) can be found below: https://www.ncbi.nlm.nih.gov/, PRJNA1209626.

## Supplementary material

Supplementary Figure 1 Geographic distribution and molecular characteristics of CRKP isolates in South Korea. The map of the Korean Peninsula illustrates the locations of regions **A–I**, where CRKP and non-CRKP isolates were collected. The pie chart displays the proportion of CRKP and non-CRKP isolates across all regions. The bar graph shows the distribution of CRKP sequence types (STs) identified in each region **(A–I)**.

## References

[B1] Argimón S. Abudahab K. Goater R. J. E. Fedosejev A. Bhai J. Glasner C. (2016). Microreact: Visualizing and sharing data for genomic epidemiology and phylogeography. *Microb. Genom.* 2:e000093. 10.1099/mgen.0.000093 28348833 PMC5320705

[B2] Bachman M. A. Oyler J. E. Burns S. H. Caza M. Lépine F. Dozois C. M. (2011). *Klebsiella pneumoniae* yersiniabactin promotes respiratory tract infection through evasion of lipocalin 2. *Infect. Immun.* 79 3309–3316. 10.1128/IAI.05114-11 21576334 PMC3147564

[B3] Bankevich A. Nurk S. Antipov D. Gurevich A. A. Dvorkin M. Kulikov A. S. (2012). SPAdes: A new genome assembly algorithm and its applications to single-cell sequencing. *J. Comput. Biol.* 19 455–477. 10.1089/cmb.2012.0021 22506599 PMC3342519

[B4] Bialek-Davenet S. Criscuolo A. Ailloud F. Passet V. Jones L. Delannoy-Vieillard A. S. (2014). Genomic definition of hypervirulent and multidrug-resistant *Klebsiella pneumoniae* clonal groups. *Emerg. Infect. Dis.* 20 1812–1820. 10.3201/eid2011.140206 25341126 PMC4214299

[B5] Bratu S. Landman D. Haag R. Recco R. Eramo A. Alam M. (2005). Rapid spread of carbapenem-resistant *Klebsiella pneumoniae* in New York City: A new threat to our antibiotic armamentarium. *Arch. Intern. Med.* 165 1430–1435. 10.1001/archinte.165.12.1430 15983294

[B6] Carattoli A. (2009). Resistance plasmid families in *Enterobacteriaceae*. *Antimicrob. Agents Chemother.* 53 2227–2238. 10.1128/AAC.01707-08 19307361 PMC2687249

[B7] Clinical and Laboratory Standards Institute (CLSI) (2019). *Performance standards for antimicrobial susceptibility testing. 29th Edn*. CLSI Supplement M100. Wayne, PA: CLSI.

[B8] Effah C. Y. Sun T. Liu S. Wu Y. (2020). *Klebsiella pneumoniae*: An increasing threat to public health. *Ann. Clin. Microbiol. Antimicrob.* 19:1. 10.1186/s12941-019-0343-8 31918737 PMC7050612

[B9] Gan R. Zhou F. X. Si Y. Yang H. Chen C. Ren C. (2022). DBSCAN-SWA: An integrated tool for rapid prophage detection and annotation. *Front. Genet.* 13:885048. 10.3389/fgene.2022.885048 35518360 PMC9061938

[B10] Gardner S. N. Slezak T. Hall B. G. (2015). kSNP3.0: SNP detection and phylogenetic analysis of genomes without genome alignment or reference genome. *Bioinformatics* 31 2877–2878. 10.1093/bioinformatics/btv271 25913206

[B11] Gupta S. K. Padmanabhan B. R. Diene S. M. Lopez-Rojas R. Kempf M. Landraud L. (2014). ARG-ANNOT, a new bioinformatic tool to discover antibiotic resistance genes in bacterial genomes. *Antimicrob. Agents Chemother.* 58 212–220. 10.1128/AAC.01310-13 24145532 PMC3910750

[B12] Hall M. B. (2022). Rasusa: Randomly subsample sequencing reads to a specified coverage. *J. Open Source Softw.* 7:3941. 10.21105/joss.03941

[B13] Han J. H. Goldstein E. J. C. Wise J. Bilker W. B. Tolomeo P. Lautenbach E. (2017). Epidemiology of carbapenem-resistant *Klebsiella pneumoniae* in a network of long-term acute care hospitals. *Clin. Infect. Dis.* 64 839–844. 10.1093/cid/ciw856 28013258 PMC5399931

[B14] Jeong S. Lee N. Park M. J. Jeon K. Kim H. S. Kim H. S. (2022). Genotypic distribution and antimicrobial susceptibilities of carbapenemase-producing *Enterobacteriaceae* isolated from rectal and clinical samples in Korean University hospitals between 2016 and 2019. *Ann. Lab. Med.* 42 36–46. 10.3343/alm.2022.42.1.36 34374347 PMC8368229

[B15] Jünemann S. Sedlazeck F. J. Prior K. Albersmeier A. John U. Kalinowski J. (2013). Updating benchtop sequencing performance comparison. *Nat. Biotechnol.* 31 294–296. 10.1038/nbt.2522 23563421

[B16] Kim M. K. Joo S. Shin E. Kim J. Yoo J. (2023). Characteristics of carbapenem-resistant *Enterobacteriaceae* (CRE) isolated in Korea, 2021. *Public Health Wkly. Rep.* 16 1–12. 10.56786/PHWR.2023.16.18.1

[B17] Kim M. N. Yong D. An D. Lee K. Chong Y. Jeong S. H. (2010). Nosocomial clustering of KPC-2-producing *Klebsiella pneumoniae* isolates in Korea. *Antimicrob. Agents Chemother*. 54, 3079–3082. 10.1128/AAC.00251-1020498310

[B18] Krawczyk P. S. Lipinski L. Dziembowski A. (2018). PlasFlow: Predicting plasmid sequences in metagenomic data using genome signatures. *Nucleic Acids Res.* 46:e35. 10.1093/nar/gkx1321 29346586 PMC5887522

[B19] Lam M. M. C. Wick R. R. Judd L. M. Holt K. E. (2021). Insights into virulence and resistance from the complex mobilome of *Klebsiella pneumoniae*. *Nat. Rev. Microbiol.* 19 211–225. 10.1038/s41579-020-00449-833067570

[B20] Lee C. R. Lee J. H. Park K. S. Kim Y. B. Jeong B. C. Lee S. H. (2016). Global dissemination of carbapenemase-producing *Klebsiella pneumoniae*: Epidemiology, genetic context, treatment options, and detection methods. *Front. Microbiol.* 7:895. 10.3389/fmicb.2016.00895 27379038 PMC4904035

[B21] Lee H. Yoon E. J. Kim D. Jeong S. H. Shin J. H. Shin J. H. (2018). Establishment of the South Korean national antimicrobial resistance surveillance system, Kor-GLASS, in 2016. *Eurosurveillance* 23:1700734. 10.2807/1560-7917.ES.2018.23.42.1700734 30352643 PMC6199867

[B22] Li J. Li Y. Song N. Chen Y. (2020). Risk factors for carbapenem-resistant *Klebsiella pneumoniae* infection: A meta-analysis. *J. Glob. Antimicrob. Resist.* 21 306–313. 10.1016/j.jgar.2019.09.006 31525540

[B23] Lim J. Sim J. Lee H. Hyun J. Lee S. Park S. (2024). Characteristics of carbapenem-resistant *Enterobacteriaceae* (CRE) in the Republic of Korea, 2022. *Public Health wkly. Rep.* 17 121–127. 10.1108/PIJPSM-04-2024-202 PMC12479968 41332463

[B24] Long S. W. Olsen R. J. Eagar T. N. Beres S. B. Zhao P. Davis J. J. (2017). Population genomic analysis of extended-spectrum β-lactamase-producing *Klebsiella pneumoniae* isolates. *mBio* 8:e00489-17. 10.1128/mBio.00489-17 28512093 PMC5433097

[B25] Mabel B. Zhang L. Ji J. (2024). Epidemiology and resistance patterns of CRKP in ICU patients. *Infect. Drug Resist.* 16 2813–2827. 10.1002/cpt.3316 37193299 PMC10182806

[B26] McArthur A. G. Waglechner N. Nizam F. Yan A. Azad M. A. Baylay A. J. (2013). The comprehensive antibiotic resistance database. *Antimicrob. Agents Chemother.* 57 3348–3357. 10.1128/AAC.00419-13 23650175 PMC3697360

[B27] Nordmann P. Cuzon G. Naas T. (2009). The real threat of *Klebsiella pneumoniae* carbapenemase-producing bacteria. *Lancet Infect. Dis.* 9 228–236. 10.1016/S1473-3099(09)70054-4 19324295

[B28] Park D. J. Yu J. K. Park K. G. Park Y. J. (2015). Genotypes of ciprofloxacin-resistant *Klebsiella pneumoniae* in Korea and their characteristics according to the genetic lineages. *Microbial Drug Resist.* 21 622–630. 10.1089/mdr.2015.0001 26207318

[B29] Peirano G. Chen L. Kreiswirth B. N. Pitout J. D. D. (2020). Emerging antimicrobial-resistant high-risk *Klebsiella pneumoniae* clones ST307 and ST147. *Antimicrob. Agents Chemother.* 64:e01148-20. 10.1128/AAC.01148-20 32747358 PMC7508593

[B30] Pellow D. Mizrahi I. Shamir R. (2020). PlasClass improves plasmid sequence classification. *PLoS Comput. Biol.* 16:e1007781. 10.1371/journal.pcbi.1007781 32243433 PMC7159247

[B31] Pitout J. D. D. Nordmann P. Poirel L. (2015). Carbapenemase-producing *Klebsiella pneumoniae*, a key pathogen set for global nosocomial dominance. *Antimicrob. Agents Chemother.* 59 5873–5884. 10.1128/AAC.01019-15 26169401 PMC4576115

[B32] Podschun R. Ullmann U. (1998). Klebsiella spp. as nosocomial pathogens: Epidemiology, taxonomy, typing methods, and pathogenicity factors. *Clin. Microbiol. Rev.* 11 589–603. 10.1128/CMR.11.4.589 9767057 PMC88898

[B33] Qi Y. Wei Z. Ji S. Du X. Shen P. Yu Y. (2011). ST11, the dominant clone of KPC-producing *Klebsiella pneumoniae* in China. *J. Antimicrob. Chemother.* 66 307–312. 10.1093/jac/dkq431 21131324

[B34] Rhee J. Y. Park Y. K. Shin J. Y. Choi J. Y. Lee M. Y. Peck K. R. (2010). KPC-producing extreme drug-resistant *Klebsiella pneumoniae* isolate from a patient with diabetes mellitus and chronic renal failure on hemodialysis in South Korea. *Antimicrob. Agents Chemother.* 54 2278–2279. 10.1128/AAC.00011-10 20211897 PMC2863605

[B35] Schwaber M. J. Klarfeld-Lidji S. Navon-Venezia S. Schwartz D. Leavitt A. Carmeli Y. (2008). Predictors of carbapenem-resistant *Klebsiella pneumoniae* acquisition among hospitalized adults and effect of acquisition on mortality. *Antimicrob. Agents Chemother.* 52 1028–1033. 10.1128/AAC.01020-07 18086836 PMC2258527

[B36] Shropshire H. A. Aitken S. L. Pincus N. B. Tariq S. A. Marschall J. (2022). Emergence and spread of *Klebsiella pneumoniae* ST307 with reduced susceptibility to carbapenems in the United States. *mBio* 13:e00497-22. 10.1128/mbio.00497-22 35357213

[B37] Siguier P. Perochon J. Lestrade L. Mahillon J. Chandler M. (2006). ISfinder: The reference centre for bacterial insertion sequences. *Nucleic Acid Res.* 34 D32–D36. 10.1093/nar/gkj014 16381877 PMC1347377

[B38] Wang F. Zou X. Zhou B. Yin T. Wang P. (2023). Clinical characteristics of carbapenem-resistant *Klebsiella pneumoniae* infection/colonisation in the intensive care unit: A 9-year retrospective study. *BMJ Open* 13:e065786. 10.1136/bmjopen-2022-065786 37308270 PMC10277079

[B39] Wyres K. L. Hawkey J. Hetland M. A. K. Fostervold A. Wick R. R. Judd L. M. (2019). Emergence and rapid global dissemination of CTX-M-15-associated *Klebsiella pneumoniae* strain ST307. *J. Antimicrob. Chemother.* 74 577–581. 10.1093/jac/dky492 30517666 PMC6376852

[B40] Wyres K. L. Lam M. M. C. Holt K. E. (2020). Population genomics of *Klebsiella pneumoniae*. *Nat. Rev. Microbiol.* 18 344–359. 10.1038/s41579-019-0315-1 32055025

[B41] Yoo J. S. Kim H. M. Yoo J. I. Yang J. W. Kim H. S. Chung G. T. (2013). Detection of clonal KPC-2-producing *Klebsiella pneumoniae* ST258 in Korea during nationwide surveillance in 2011. *J. Med. Microbiol.* 62 1338–1342. 10.1099/jmm.0.059428-0 23741020

[B42] Yoon E. J. Yang J. W. Kim J. O. Lee H. Lee K. J. Jeong S. H. (2018a). Carbapenemase-producing *Enterobacteriaceae* in South Korea: A report from the National Laboratory Surveillance System. *Future Microbiol.* 13 771–783. 10.2217/fmb-2018-0022 29478336

[B43] Yoon E. J. Kim J. O. Kim D. Lee H. Yang J. W. Lee K. J. (2018b). *Klebsiella pneumoniae* carbapenemase producers in South Korea between 2013 and 2015. *Front. Microbiol.* 9:56. 10.3389/fmicb.2018.00056 29422888 PMC5788937

