# Corrigendum: Genomic analysis of carbapenem-resistant *Klebsiella pneumoniae* blood isolates from nationwide surveillance in South Korea

**DOI:** 10.3389/fmicb.2025.1627539

**Published:** 2025-06-11

**Authors:** Younggwon On, Jung Wook Kim, Juyoung Lee, Jung Sik Yoo

**Affiliations:** ^1^Division of Antimicrobial Resistance Research, Center for Infectious Disease, National Institute of Health, Korea, Cheongju, South Korea; ^2^Division of Zoonotic and Vector Borne Diseases Research, Center for Infectious Disease, National Institute of Health, Korea, Cheongju, South Korea; ^3^Division of Biobank for Health Sciences, Department of Future Healthcare, National Institute of Health, Korea, Cheongju, South Korea

**Keywords:** antibiotic resistance, whole-genome sequencing, nationwide, surveillance, carbapenem, genomic epidemiology

In the published article, there was an error in Figures 1, 3, 4, 5, and their respective captions, as published. Figures 1, 3, 4, 5, as used in the published article, were not the latest iterations of these figures.

**Figure 1:**
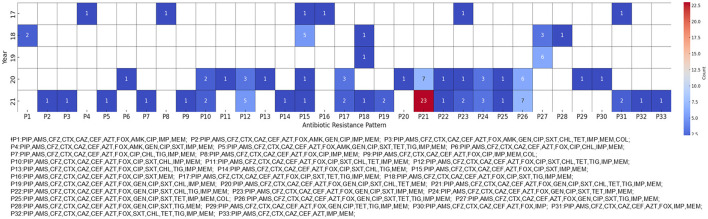
Antibiotic resistance patterns in carbapenem-resistant *Klebsiella pneumoniae* (CRKP) isolates. The heatmap shows the antibiotic resistance patterns of CRKP isolates from 2017 to 2021. Each cell represents the number of isolates exhibiting a particular resistance pattern, with darker red shades indicating higher numbers of isolates. For readability, the patterns are abbreviated and matched to arbitrary code.

**Figure 3:**
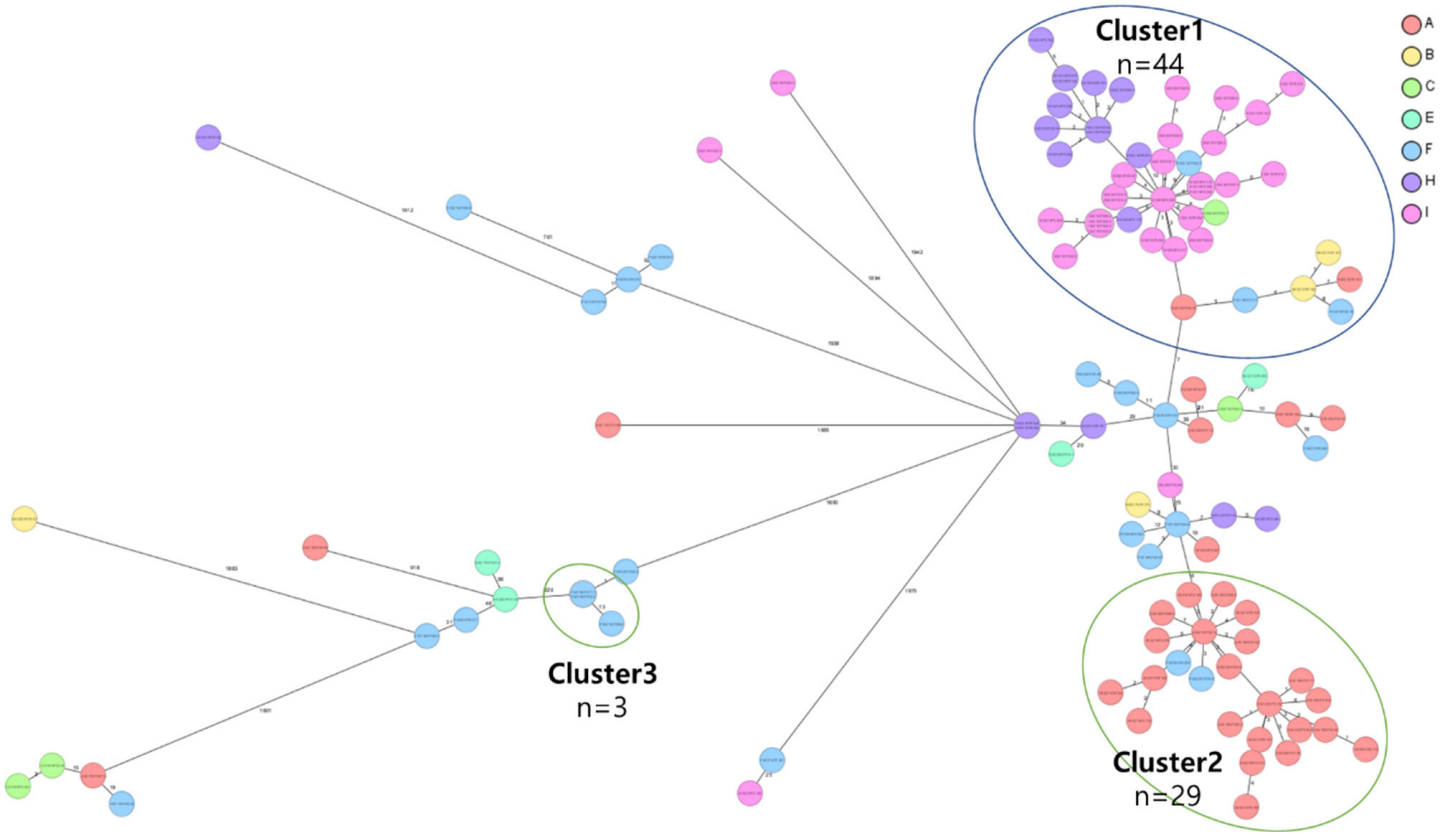
Core genome multi-locus sequence type (cgMLST) analysis of carbapenem-resistant *Klebsiella pneumoniae* (CRKP) isolates from the nine region hospitals in Korea. The isolates were analyzed using cgMLST in Ridom SeqSphere+ and were visualized in a minimum spanning tree. Isolates with 12 allele differences between them were grouped together in clusters with the isolates per cluster shown in circles. The number of different alleles between clusters and unique isolates is shown on the connecting lines (not to scale).

**Figure 4:**
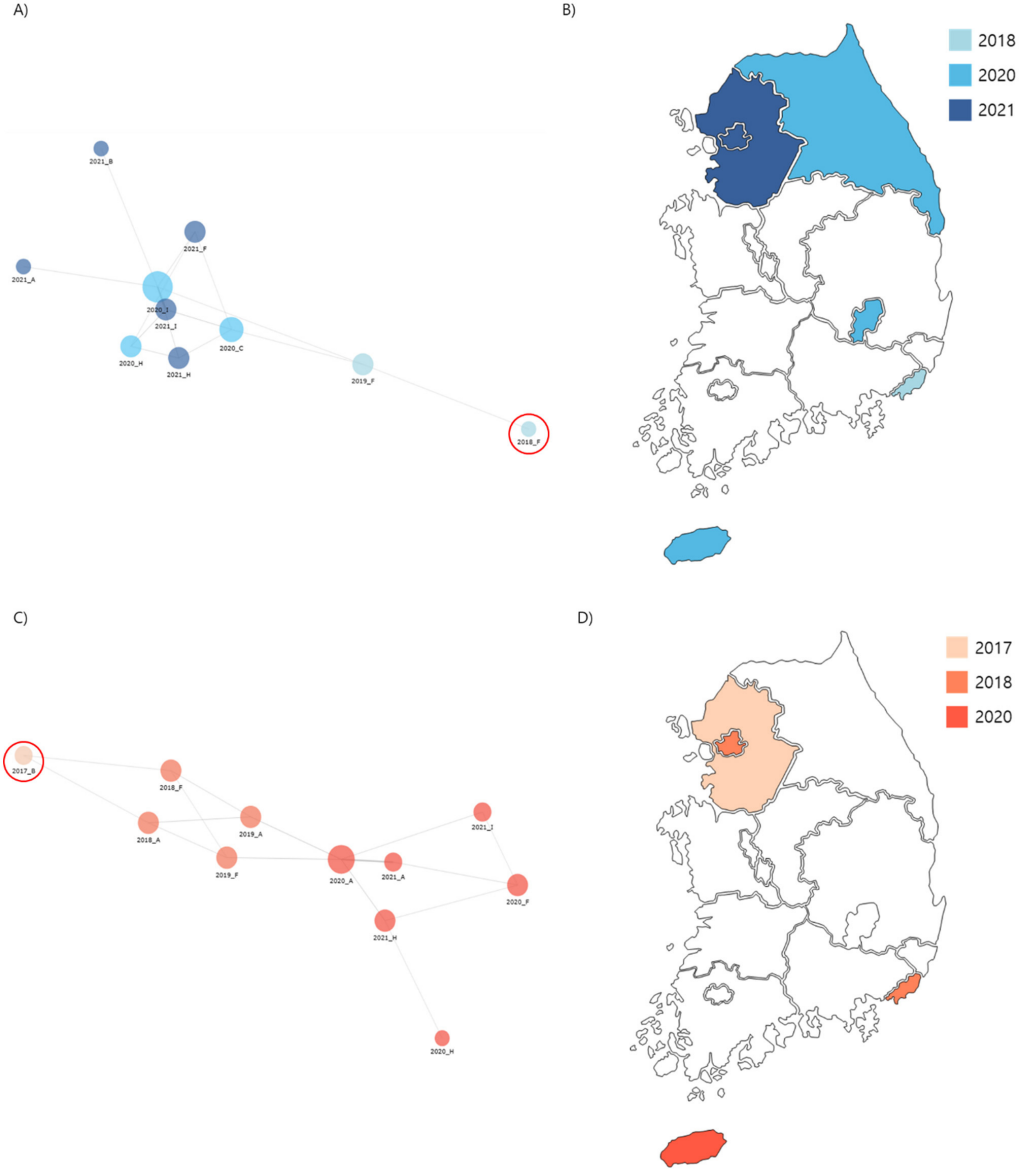
Phylogenetic analysis of carbapenem-resistant *Klebsiella pneumoniae* (CRKP) isolates from ST307 Cluster 1 and 2. **(A)** A core-SNP tree and cgMLST analysis were used to visualize isolate relationships, with a node-link diagram highlighting Cluster 1 proximity. **(B)** Spread routes of strains in Cluster 1 by region. **(C)** A node-link diagram visualizing isolated strains in Cluster 2, with the same layout and parameters as **(A)**. **(D)** Spread routes of strains in Cluster 2 by region, using the same visualization style as **(B)**.

**Figure 5:**
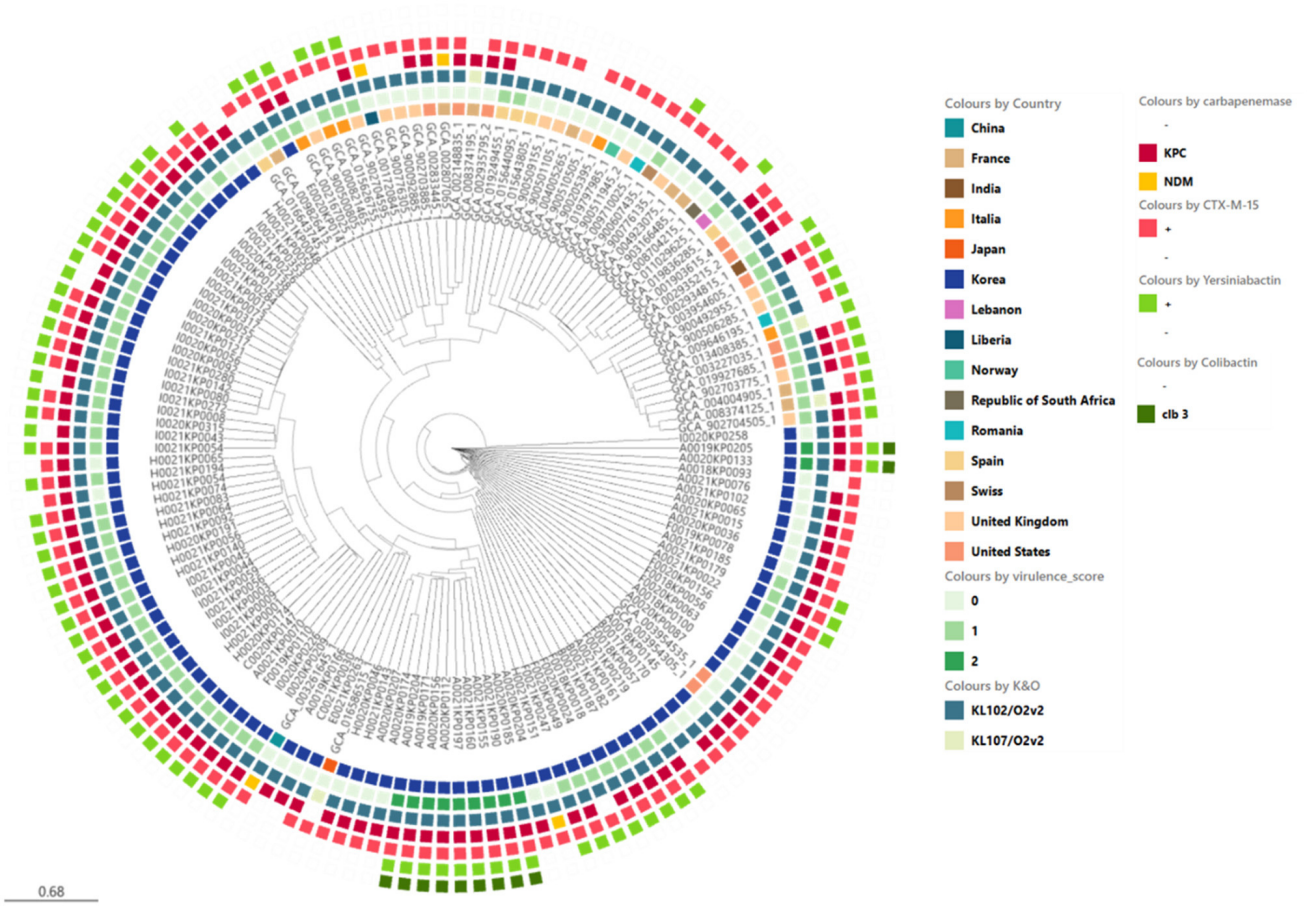
Phylogenetic analysis of ST307 *Klebsiella pneumoniae* isolates including domestic and global reference genomes. The SNP-based neighbor joining (kSNP4) phylogenetic tree was generated. *KPC* gene and VF genes are indicated.

The corrected figures, in addition to their corrected captions, appear below.

In the published article, there were numerous textual errors in **Section 3.2, “Characteristics of CRKP isolates**.”

A correction has been made to the following sentences within Section 3.2:

1. “The most prevalent pattern, designated P4, exhibited resistance to antibiotics belonging to the penicillin, cephalosporin, carbapenem, monobactam, aminoglycoside, fluoroquinolone, tetracycline, and sulfonamide classes.”
2. “The P4 pattern was first identified in 2018 and showed a marked increase by 2021 (Figure 1).”
3. “This pattern was initially detected in Region A in 2017 and was confirmed to be confined to this region until 2019.”
4. “By 2021, the presence of the P4 pattern had been further verified in a hospital in Region I, which participated in the Kor-GLASS surveillance system for the first time.”

The corrected sentences appear below:

1. “The most prevalent pattern, designated P21, exhibited resistance to antibiotics belonging to the penicillin, cephalosporin, carbapenem, monobactam, aminoglycoside, fluoroquinolone, tetracycline, tigecycline, and sulfonamide classes.”
2. “The P21 pattern was first identified in 2018 and showed a marked increase by 2021 (Figure 1).”
3. “This pattern was initially detected in Region A in 2017 and was confirmed to be confined to this region until 2019.”
4. “By 2021, the presence of the P21 pattern had been further verified in a hospital in Region I, which participated in the Kor-GLASS surveillance system for the first time.”

The authors apologize for these errors and state that they do not change the scientific conclusions of the article in any way. The original article has been updated.

