# Supplementary figures and images for: Genomic analysis of carbapenem-resistant Klebsiella pneumoniae blood isolates from nationwide surveillance in South Korea
Source: Front Microbiol. 2025 May 6;16:1562222. doi: 10.3389/fmicb.2025.1562222 (PMC12089144; doi:10.3389/fmicb.2025.1562222)

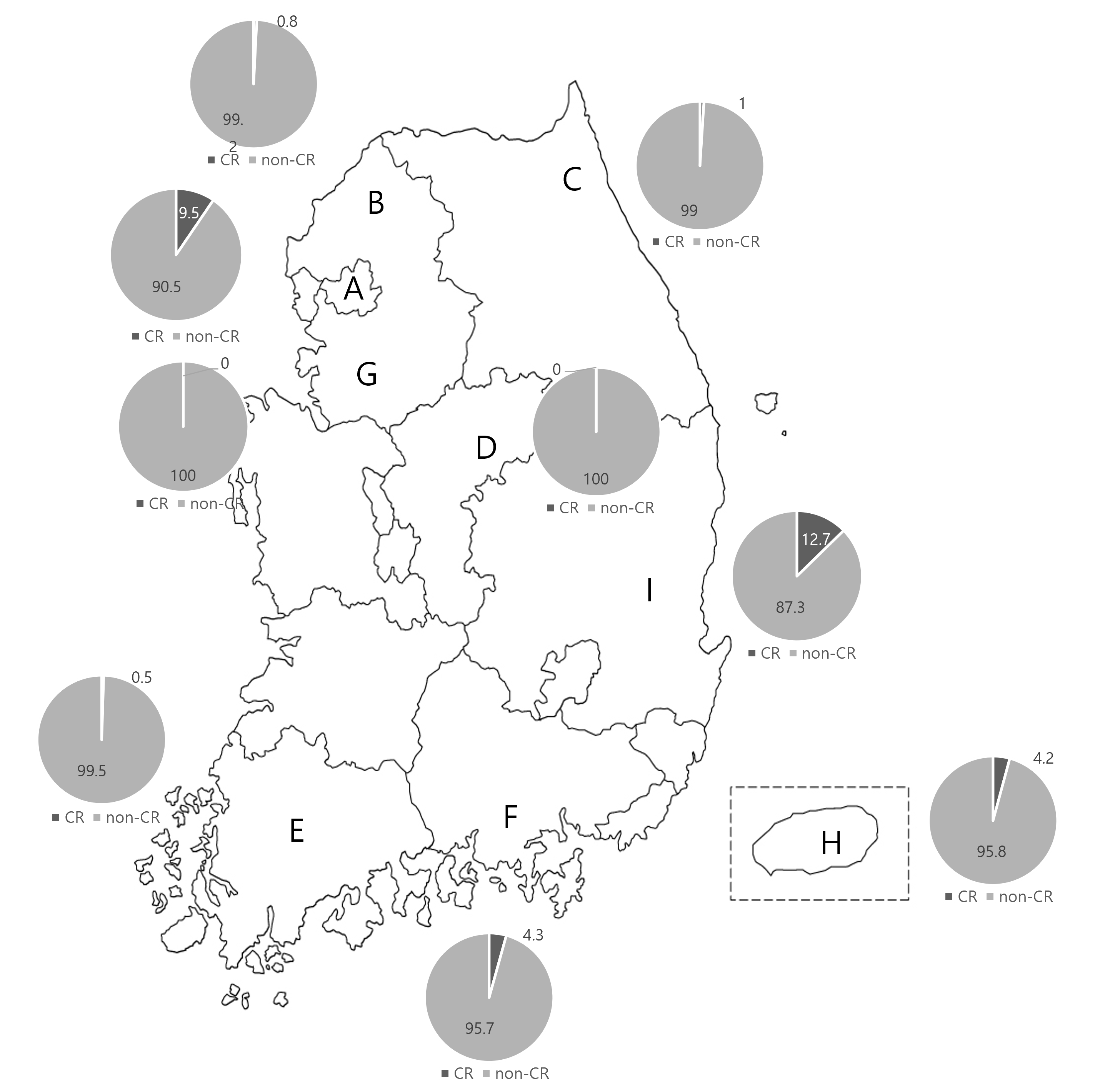

Supplement: Supplementary Figure 1 — Geographic distribution and molecular characteristics of CRKP isolates in South Korea. The map of the Korean Peninsula illustrates the locations of regions A–I, where CRKP and non-CRKP isolates were collected. The pie chart displays the proportion of CRKP and non-CRKP isolates across all regions. The bar graph shows the distribution of CRKP sequence types (STs) identified in each region (A–I). [file Image_1.tif]
